# Supplementary material for: Health status of Polychrus gutturosus based on physical examination, hematology and biochemistry parameters in Costa Rica
Source: PeerJ. 2021 Jan 6;9:e10649. doi: 10.7717/peerj.10649 (PMC7796665; doi:10.7717/peerj.10649)
Supplement: Supplemental Information 1 — Saurian hematology and biochemistry for comparison. [file peerj-09-10649-s001.docx]

**Supplementary data**

Health status of the Berthold’s bush anole (*Polychrus gutturosus*) based on physical examination, hematology and biochemistry parameters in Costa Rica

Randall Arguedas, Lizbeth Ovares, Viviana P. Arguedas, Rodolfo Vargas, Marco D. Barquero

List of published papers including data on hematology and/or biochemistry of iguanian lizards. Order is by family (following phylogenetic relationships according to Pyron et al. [2013]) and by year.

**Chamaeleonidae**

Molina-Prescott I, López R, Molleda JM, Martín E, Quevedo MA, Díaz-Paniagua C, Cuadrado M (2001) Hematología y bioquímica sanguínea en el camaleón común (*Chamaeleo chamaeleon*). In: Libro de Resúmenes del VII Congreso Anual. Sociedad Española de Medicina Interna Veterinaria, Las Palmas de Gran Canaria, pp 17–23.

Laube A, Pendl H, Clauss M, Altherr B, Hatt JM (2016) Plasma biochemistry and hematology reference values of captive panther chameleons (*Furcifer pardalis*) with special emphasis on seasonality and gender differences. *J Zoo Wildl Med* 47:743–753.

**Agamidae**

Sodeinde OA, Ogunjobi AA (1994) Haematological values of the rainbow lizard *Agama agama* L. *Herpetol J* 4:86–90.

Ellman M (1997) Hematology and plasma chemistry of the inland bearded dragon, *Pogona vitticeps*. *Bull Assoc Reptilian Amphibian Vet* 7:10–12.

Said KM, Hussein HK (2001) Variations in some haematological parameters and gonadal indices of *Uromastyx aegyptius* during hibernation and active periods. *Egypt J Biol* 3:81–85.

Mayer J, Knoll J, Innis C, Mitchell MA (2005) Characterizing the hematologic and plasma chemistry profiles of captive Chinese water dragons, *Physignathus cocincinus*. *J Herpetol Med Surg* 15:45–52.

Pal A, Parida SP, Swain MM (2008) Hematological and plasma biochemistry in fan throated lizard *Sitana ponticeriana* (Sauria: Agamidae). *Russ J Herpet* 15:110–116.

Naldo JL, Libanan NL, Samour JH (2009) Health assessment of a spiny-tailed lizard (*Uromastyx* spp.) population in Abu Dhabi, United Arab Emirates. *J Zoo Wildl Med* 40:445–452.

Gül Ç, Tosunoğlu M (2011) Hematological reference intervals of four agamid lizard species from Turkey (Squamata: Sauria: Agamidae). *Herpetozoa* 24:51–59.

Parida SP, Dutta SK, Pal A (2012) Hematological and plasma biochemistry in *Psammophilus blanfordanus* (Sauria: Agamidae). *Comp Clin Path* 21:1387–1394.

Johnson RSP, Harlow PS, Phillips CA, Hall EJS (2018) Baseline morphometric, haematological and plasma biochemical parameters in free-ranging eastern water dragons (*Intellagama lesueurii lesueurii*). *Aust Vet J* 96:450–457.

**Tropiduridae**

Scorza JV (1971) Some haematological observations on *Tropidurus torquatus* (Sauria, Iguanidae) from Venezuela. *J Zool* 165:557–561.

Arguedas R, Steinberg D, Lewbart GA, Deresienski D, Lohmann KJ, Muñoz-Pérez JP, Valle CA (2018) Haematology and biochemistry of the San Cristóbal lava lizard (*Microlophus bivittatus*). *Conserv Physiol* 6, coy046. doi.org/10.1093/conphys/coy046.

**Iguanidae**

Acuña ML (1974) The hematology of the tropical lizard *Iguana iguana* Linnaeus: II. Seasonal variations. Herpetologica 30:299–303.

Divers SJ, Redmayne G, Aves EK (1996) Haematological and biochemical values of 10 green iguanas (*Iguana iguana*). *Vet Rec* 138:203–205.

Alberts AC, Oliva ML, Worley MB, Telford Jr SR, Morris PJ, Janssen DL (1998) The need for pre-release health screening in animal translocations: a case study of the Cuban iguana (*Cyclura nubila*). *Anim Conserv* 1:165–172.

Harr KE, Alleman AR, Dennis PM, Maxwell LK, Lock BA, Bennett RA, Jacobson ER (2001) Morphologic and cytochemical characteristics of blood cells and hematologic and plasma biochemical reference ranges in green iguanas. *J Am Vet Med Assoc* 218:915–921.

James SB, Iverson J, Greco V, Raphael BL (2006) Health assessment of Allen Cays rock iguana, *Cyclura cychlura inornata*. *J Herpetol Med Surg* 16:93–98.

Maria R, Ramer J, Reichard T, Tolson PJ, Christopher MM (2007) Biochemical reference intervals and intestinal microflora of free-ranging Ricord's iguanas (*Cyclura ricordii*). *J Zoo Wildl Med* 38:414–419.

Novoa-Fajardo D, Benitez-Tumay I, Corredor-Matus JR, Rodriguez-Pulido J (2008) Hallazgos hematológicos en iguana verde Suramericana (*Iguana iguana*), de ejemplares ubicados en zona urbana y suburbana de villavicencio (Meta). *Orinoquia* 12:67–79.

Lewbart GA, Hirschfeld M, Brothers JR, Muñoz-Pérez JP, Denkinger J, Vinueza L, García J, Lohmann KJ (2015) Blood gases, biochemistry and haematology of Galápagos marine iguanas (*Amblyrhynchus cristatus*). *Conserv Physiol* 3, cov034. doi.org/10.1093/conphys/cov034.

Silveira MD, Alves JEO, Vieira EMP (2017) Parâmetros hematológicos e bioquímicos da espécie *Iguana iguana*: Revisão de literatura. *Acta Biomed Bras* 8:1–12.

Lewbart GA, Grijalva CJ, Calle PP, Ingerman K, Muñoz-Pérez, JP, Quezada G, Vera CA, Gentile G, Valle CA (2019) Health assessment of *Conolophus subcristatus*, *Conolophus pallidus*, and *C. subcristatus* X *Amblyrhynchus cristatus* hybrid (Galápagos land iguanas). *PLoS One* 14, e0222884. doi.org/10.1371/journal.pone.0222884.

**Phrynosomatidae**

González-Morales JC, Beamonte-Barrientos R, Bastiaans E, Guevara-Fiore P, Quintana E, Fajardo V (2017) A mountain or a plateau? Hematological traits vary nonlinearly with altitude in a highland lizard. *Physiol Biochem Zool: Ecol Evol Approaches* 90:638–645.

McEntire MS, Pich A, Zordan M, Barber D, Rains N, Erxleben D, Heatley JJ, Sanchez CR (2018) Hematology of free-ranging and managed Texas horned lizards (*Phrynosoma cornutum*). *J Wildl Dis* 54:802–808.

**Liolaemidae**

Ruiz G, Rosenmann M, Nuñez H (1993) Blood values in South American lizards from high and low altitudes. *Comp Biochem Physiol A Mol Integr Physiol* 106:713–718.

Ceballos de Bruno S (1995) Algunos parámetros hematológicos en *Liolaemus wiegmannii* (Sauria: Tropiduridae). Número de eritrocitos, número de leucocitos y fórmula leucocitaria. Morfología de células sanguíneas y de médula ósea. *Cuad Herpetol* 9:51–56.

**Corytophanidae**

Dallwig RK, Paul-Murphy J, Thomas C, Medlin S, Vaughan C, Sullivan L, Sladky KK, Ramirez O, Herrera G (2011) Hematology and clinical chemistry values of free-ranging basilisk lizards (*Basiliscus plumifrons*) in Costa Rica. *J Zoo Wildl Med* 42:205–213.

**Dactyloidae**

Dessauer HC (1952) Biochemical studies on the lizard, *Anolis carolinensis*. *Exp Biol Med* 80:742–744.
